# Mouse phenome database: curated data repository with interactive multi-population and multi-trait analyses

**DOI:** 10.1007/s00335-023-10014-3

**Published:** 2023-08-15

**Authors:** Molly A. Bogue, Robyn L. Ball, David O. Walton, Matthew H. Dunn, Georgi Kolishovski, Alexander Berger, Anna Lamoureux, Stephen C. Grubb, Matthew Gerring, Matthew Kim, Hongping Liang, Jake Emerson, Timothy Stearns, Hao He, Gaurab Mukherjee, John Bluis, Sara Davis, Sejal Desai, Beth Sundberg, Beena Kadakkuzha, Govindarajan Kunde-Ramamoorthy, Vivek M. Philip, Elissa J. Chesler

**Affiliations:** 1 The Jackson Laboratory, 600 Main Street, Bar Harbor, ME USA; 2 University of British Columbia, Vancouver, BC Canada

## Abstract

The Mouse Phenome Database continues to serve as a curated repository and analysis suite for measured attributes of members of diverse mouse populations. The repository includes annotation to community standard ontologies and guidelines, a database of allelic states for 657 mouse strains, a collection of protocols, and analysis tools for flexible, interactive, user directed analyses that increasingly integrates data across traits and populations. The database has grown from its initial focus on a standard set of inbred strains to include heterogeneous mouse populations such as the Diversity Outbred and mapping crosses and well as Collaborative Cross, Hybrid Mouse Diversity Panel, and recombinant inbred strains. Most recently the system has expanded to include data from the International Mouse Phenotyping Consortium. Collectively these data are accessible by API and provided with an interactive tool suite that enables users’ persistent selection, storage, and operation on collections of measures. The tool suite allows basic analyses, advanced functions with dynamic visualization including multi-population meta-analysis, multivariate outlier detection, trait pattern matching, correlation analyses and other functions. The data resources and analysis suite provide users a flexible environment in which to explore the basis of phenotypic variation in health and disease across the lifespan.

## Introduction

The Mouse Phenome Database (MPD; https://phenome.jax.org) (Bogue et al. 2023) is an NIH-recognized Biomedical Data Repository (https://sharing.nih.gov/data-management-and-sharing-policy/sharing-scientific-data/repositories-for-sharing-scientific-data) for phenotype and genotype data. Since 2001 MPD has provided researchers with a persistent public repository for data from individual mice and strains and makes it public, fulfilling NIH data sharing policies for principal investigators. Data are contributed from investigators around the world, representing studies supported by all institutes of the NIH and over one hundred funding agencies and foundations. Data are curated and annotated with community standard ontologies such as Mammalian Phenotype (MP) (Smith and Eppig 2012), Vertebrate Trait (VT) (Park et al. 2013), and Adult Mouse Anatomy (MA) (Hayamizu et al. 2005) ontologies. These attributes are related to human disease through an ongoing effort to integrate human and mouse phenotype data via dominant ontologies (Human Phenotype Ontology and Mammalian Phenotype ontology) through the Mouse-Human Ontology Mapping Initiative (Stefancsik et al. 2023). These mappings facilitate data selection and analyses that aggregate mouse data by human disease annotations. The ontology annotations and other curated metadata allow users to select and analyze relevant data using MPD analysis tools, some of which are presented in this paper. Detailed protocols are available for most datasets, or a PubMed link is provided for the accompanying publication so that users can readily access protocol information. Studies are presented on the MPD website following ARRIVE Guidelines (Animal Reporting of In Vivo Experiments) (Percie du Sert et al. 2020); fields are available for items in the recently released ARRIVE 2.0 checklist, including animal documentation (housing and husbandry), environmental parameters, and detailed procedural information (including equipment and reagents). We use Research Resource Identification Numbers (RRIDs) when possible for reagents and software (Bandrowski and Martone 2016).

MPD houses genotype data (below) and phenotype data for thousands of baseline and treatment measures, including drug studies, diet-effect studies, infectious disease challenges, toxicology studies, surgeries, and other environmental perturbations. Human disease areas benefitting from MPD include substance use disorders, cancer, immune function disorders, liver disease, reproductive conditions, bone and connective tissue disorders, neuromuscular disease, neurodegenerative disease, cardiovascular disease, endocrine/exocrine system disorders, kidney/renal disease, and respiratory disease among others. MPD can be used for many research applications, including choosing optimal strains for: modeling human disease, elucidating shared genetics, discovering genotype–phenotype relationships, formulating hypotheses and testing in silico, identifying sensitized strain backgrounds for genetic engineering, and many others.

A host of features have been added to make MPD a more FAIR-compliant (Findable, Accessible, Interoperable, and Reusable) (Wilkinson et al. 2016) and TRUST-worthy (Transparency, Responsibility, User focus, Sustainability, and Technology) (Lin et al. 2020) resource to meet current expectations for data archiving, data re-use and to make the data submission process more efficient (these updates are described in detail in Bogue et al. 2023). For example, we have migrated the full MPD ecosystem to Google Cloud Platform (GCP). These updates support traceability and reproducibility and enable interoperability with other public resources.

Here we highlight the current contents of MPD and showcase several interactive analytical tools.

## Current contents

### Phenotypic diversity

MPD houses data from reproducible strains and heterogeneous populations. Data are available for inbred, recombinant inbred, chromosome substitution, F1 hybrid, transgenic, and targeted mutant strains as well as heterogeneous populations such as the Diversity Outbred (Churchill et al. 2012) (Svenson et al. 2012), UM-HET3 (Nadon et al. 2017), and various crosses for QTL analysis including many obtained from the QTL Archive. There are over 4500 strains and populations with measurement data in MPD, representing thousands of phenotypes for behavior, anatomy, or physiology. Ontology terms have been annotated to each phenotype measure (usually multiple terms), and a data dictionary includes additional metadata about variable types, distributional characteristics, and other information needed to support exposure to appropriate analysis tools and visualization.

A major new addition is the first integration of data from the International Mouse Phenotyping Consortium (IMPC) (Peterson and Murray 2022; Groza et al. 2023) which includes the NIH Knock-out Mouse Phenotyping (KOMP) centers. Several recent studies have reported using KOMP knockout mice (Basilico et al. 2022; Brommage and Ohlsson 2019; Cacheiro et al. 2019; da Silva-Buttkus et al. 2023; Higgins et al. 2022, and many others). The IMPC consortium has characterized thousands of single-gene deletion mutations on a wide array of phenotyping assays coordinated across centers. Collectively these data provide a catalog of the effects of gene perturbations on each phenotype obtainable from the program’s web portal at https://mousephenotypes.org. The Mouse Phenome Database has ingested data from The Jackson Laboratory (JAX) KOMP center and provides standardized effect sizes across the population and traits. Placing the data in MPD complements the existing tools at the IMPC’s site by allowing users to work with a suite of multi-gene and multi-trait integrative analysis tools, to visualize and analyze sets of genes and traits, to examine individual values by genotype and sex, to find genetic perturbations that match phenotypic profiles across physiological and behavioral traits, to identify trait correlates and bivariate outliers, to compare early and late adult phenotypes, and many other statistical functions. By placing the consortium’s data in this flexible environment, many additional capabilities are provided to geneticists and disease researchers.

### Genotypic diversity

Historically MPD accessioned mouse genotype data (SNPs/INDELs) from 580 reproducible populations which includes inbred, recombinant inbred, chromosome substitution, hybrid mouse diversity panel, and Collaborative Cross strains. These datasets, however, vary tremendously in density across populations which has severely limited the collective analysis of genomic and phenomic data from these strains. The research community needed an analytical resource that harmonized these data while filling in gaps in the data with accurately imputed genotypes. The recently released GenomeMUSter (https://muster.jax.org) is a comprehensive mouse genetic variation resource that provides typed, sequenced, and imputed allelic states for 657 mouse strains at 106.8 + million genomic locations. Every strain in GenomeMUSter has allelic state data for at least 84.6 + million sites with a median coverage of 97.8 + million sites, providing a dense variant grid for analytical pipelines, e.g., cross-trait cross-population genome-wide meta-analyses. See more information about GenomeMUSter below.

## Selected tools

Below we highlight applications of the expanded interactive/integrative tool suite in MPD.

### Estimating replicability from genotype × laboratory interactions

Replicability of data from model organism studies is a longstanding challenge, requiring reproducible protocols and a means of estimating the extent to which an observation is likely to be observed across laboratories (Kafkafi et al. 2005). Benjamini and colleagues described an approach for estimating replicability of a single laboratory study, using archival data to estimate the variance of genotype by laboratory interactions (Kafkafi et al. 2017). We have implemented this approach in MPD (Jaljuli et al. 2023) so that users may enter data from an experiment and evaluate it against a selection of comparable studies executed across laboratories (not shown).

### Analysis and visualization of results from complex study designs—repeated measures

**Fig. 1:**
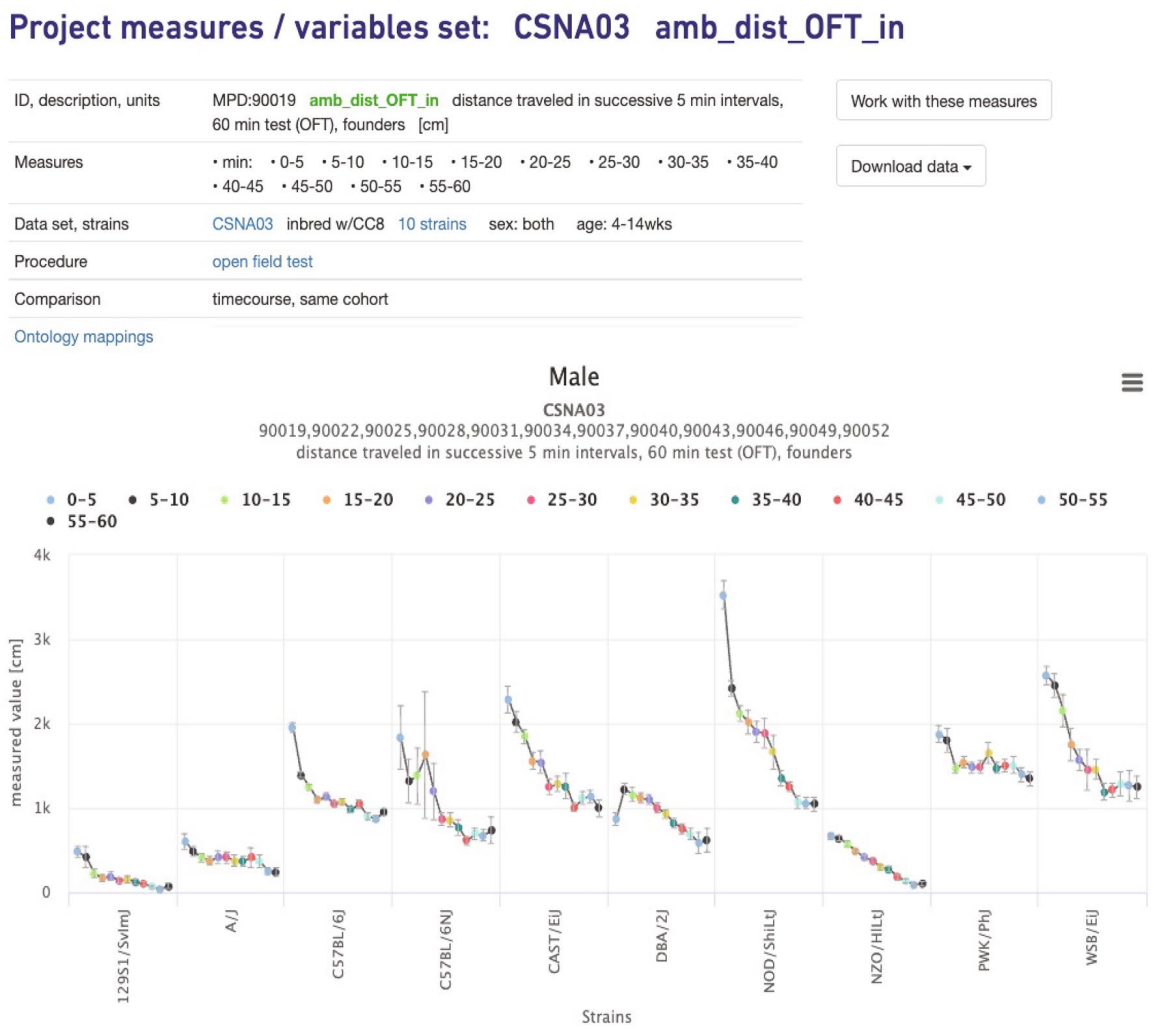
Repeated measures plot. Time course for a single cohort of Collaborative Cross founder mice. Distance traveled in the Open Field in successive 5-min intervals. *X*-axis is strain, *Y*-axis is the measured value in centimeters. CSNA03 is the MPD project symbol (Center for Systems Neurogenetics of Addiction). Data can be found at: https://phenome.jax.org/measureset/90019

MPD has a set of basic tools for analysis and visualization of each measurement endpoint, and we have expanded the set of visualizations for complex designs including repeated measures and factorial studies. Selected repeated measures can be viewed in a single plot as shown in Fig. 1. Repeated measures are those from a single cohort of animals for a time course or dose–response curve, for example. A parallel plot is also available to view the data with overlapping color-coded strain means (not shown). From here, users can access data from individual measures where the following are available: measure summary (tabular), ANOVA, Q-Q normality assessment (plot), strain means table (unadjusted and least squares mean), individual animal values, and GWAS results (Manhattan plot) using mixed-effect models if the strain set used is eligible for analysis (not shown).

### Lifespan and related phenotypes (heterogeneous population)

**Fig. 2:**
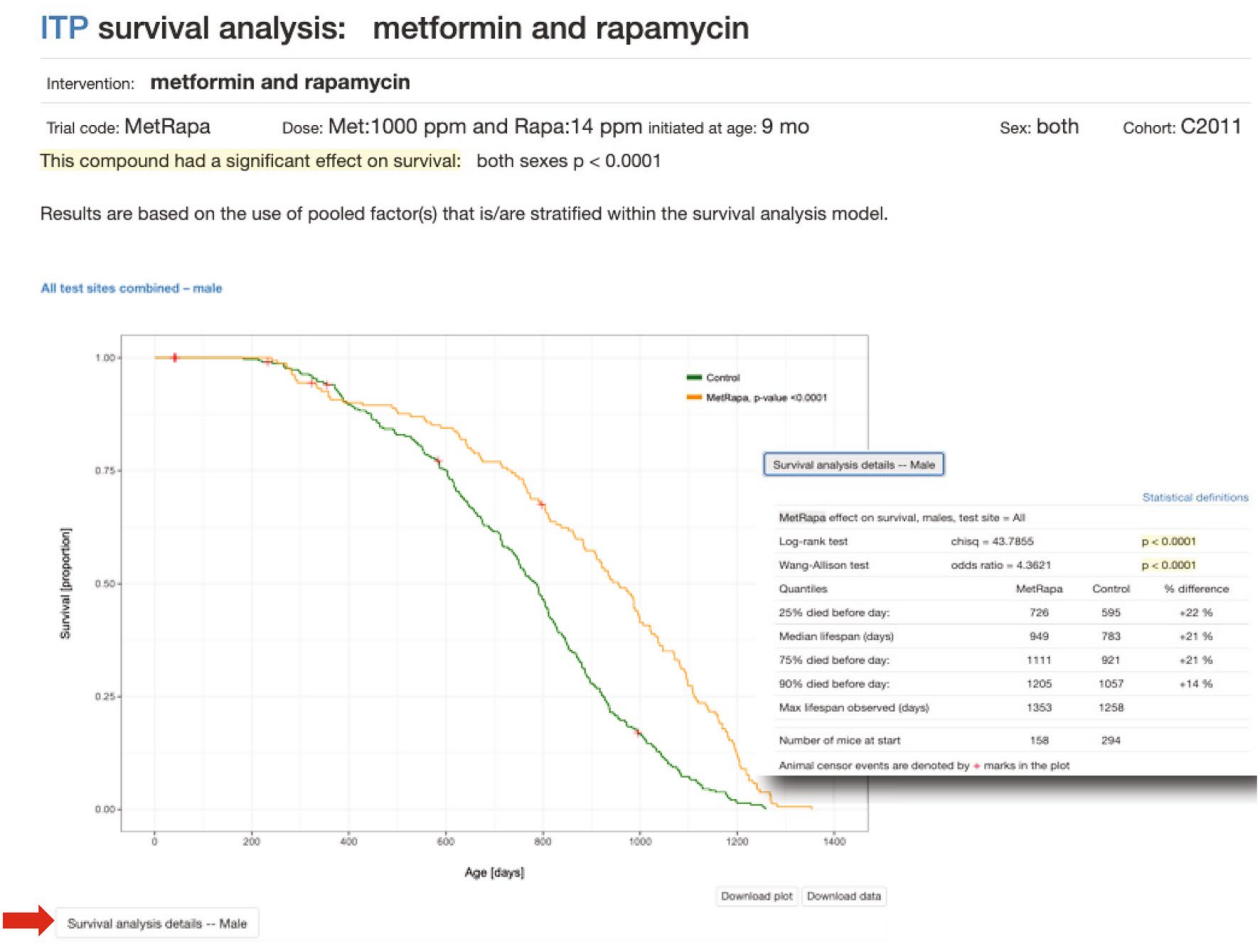
Kaplan–Meier plot of lifespan data. From the NIA supported multi-site Interventions Testing Program (ITP). The survival plot for metformin + rapamycin combined is shown for males (there is also a plot for females, not shown). Clicking on the button below the plot ‘Survival Analysis Details’ (red arrow) reveals statistical analysis data (shown in inset). Data can be found at: https://phenome.jax.org/itp/surv/MetRapa/C2011

Survival studies are another complex design, used in aging and other application areas to evaluate strain differences in longevity or response to exposures. Kaplan–Meier plots are available for survival data as shown in Fig. 2. Statistical analysis results are also provided including p-values (inset). Plots and analyses are available for aging-related phenotypes as well such as body weight, grip strength, and rotarod (not shown).

### Correlation matrix

**Fig. 3:**
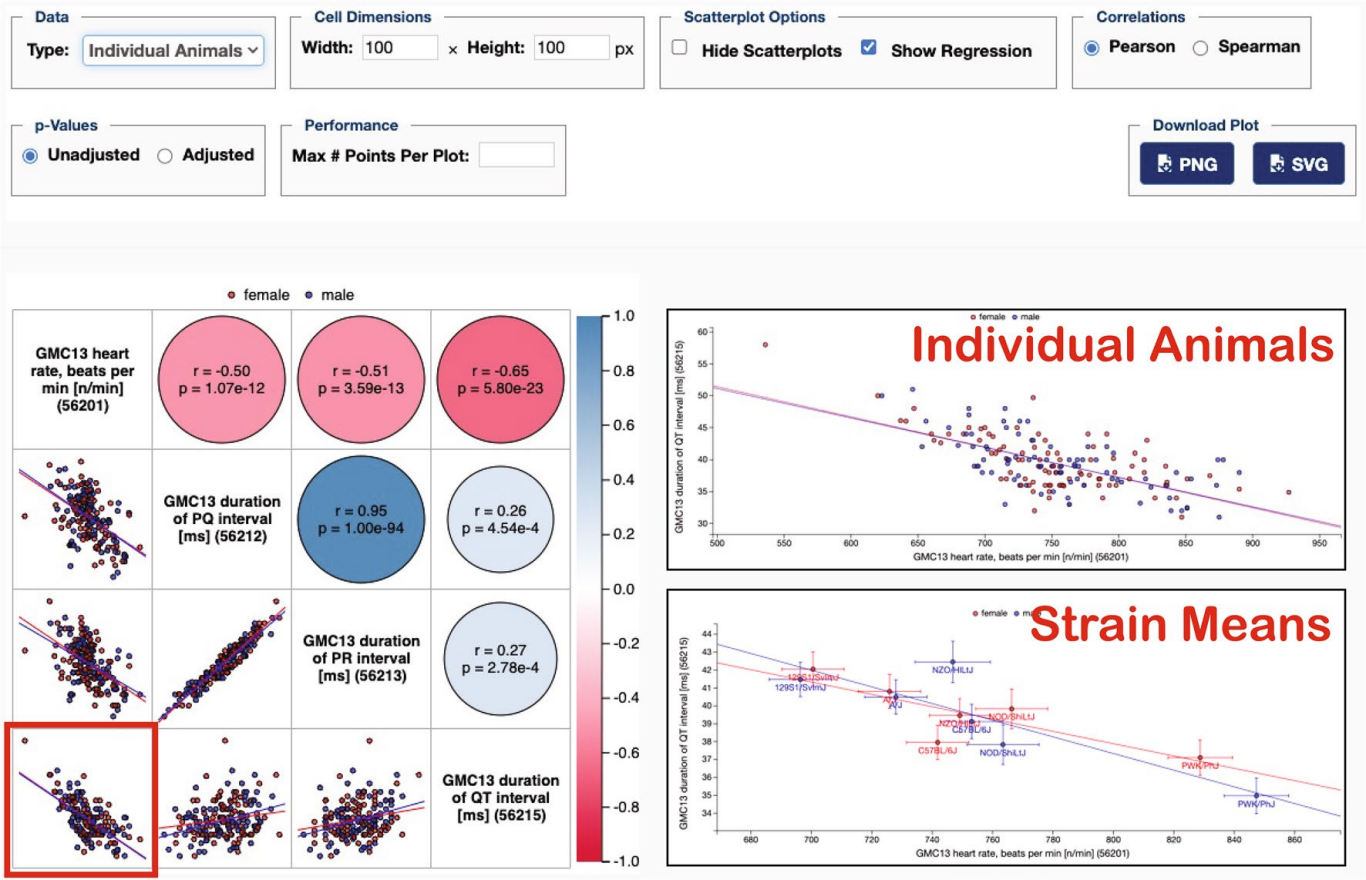
Scatterplots and correlations tool. Applied to German Mouse Clinic data for Collaborative Cross founder strains for ECG data. Users may view Pearson or Spearman correlations (see control panel above plot on the right). Thumbnail scatterplots lie beneath the diagonal (red, female; blue, male), and above the diagonal are color-coded correlation coefficients (the more intense the color, the higher the absolute value of the correlation coefficient) with circle size corresponding to p-values (the lower the p-value, the larger the circle). The red square on the lower-left cell indicates that we have clicked on that cell. From there we can get enlarged scatterplots as shown in the right insets for individual animals and for strain means. Data can be found at: https://phenome.jax.org/measures/56201, https://phenome.jax.org/measures/56212, https://phenome.jax.org/measures/56213, https://phenome.jax.org/measures/56215

Selected measures can be viewed in a single matrix with a number of viewing options as shown in Fig. 3. This tool enables the elucidation of shared genetics through the identification of correlated pairwise measures. Strain means or individual animals (shown as insets) can be analyzed. Below the diagonal of the matrix are thumbnail scatterplots of the pairwise measures shown, and above the diagonal are color-coded circles indicating the strength of the correlation (the more intense the color, the higher the absolute value of the correlation coefficient), and size of the circle indicating the p-value (the lower the p-value, the larger the circle). Any cell in the matrix is clickable. Clicking on cells along the diagonal takes users to a plot of the measurement indicated. Clicking on either side of the diagonal, for example, clicking on the red-outlined cell takes users to an enlarged scatterplot (upper inset) along with a table showing correlation coefficients and p-values for both Pearson and Spearman analyses (not shown). Plot options include the ability to show strain means (instead of individual animals) with error bars and with labels (strain names) on those data points (lower inset).

### Multivariate outlier detection

**Fig. 4:**
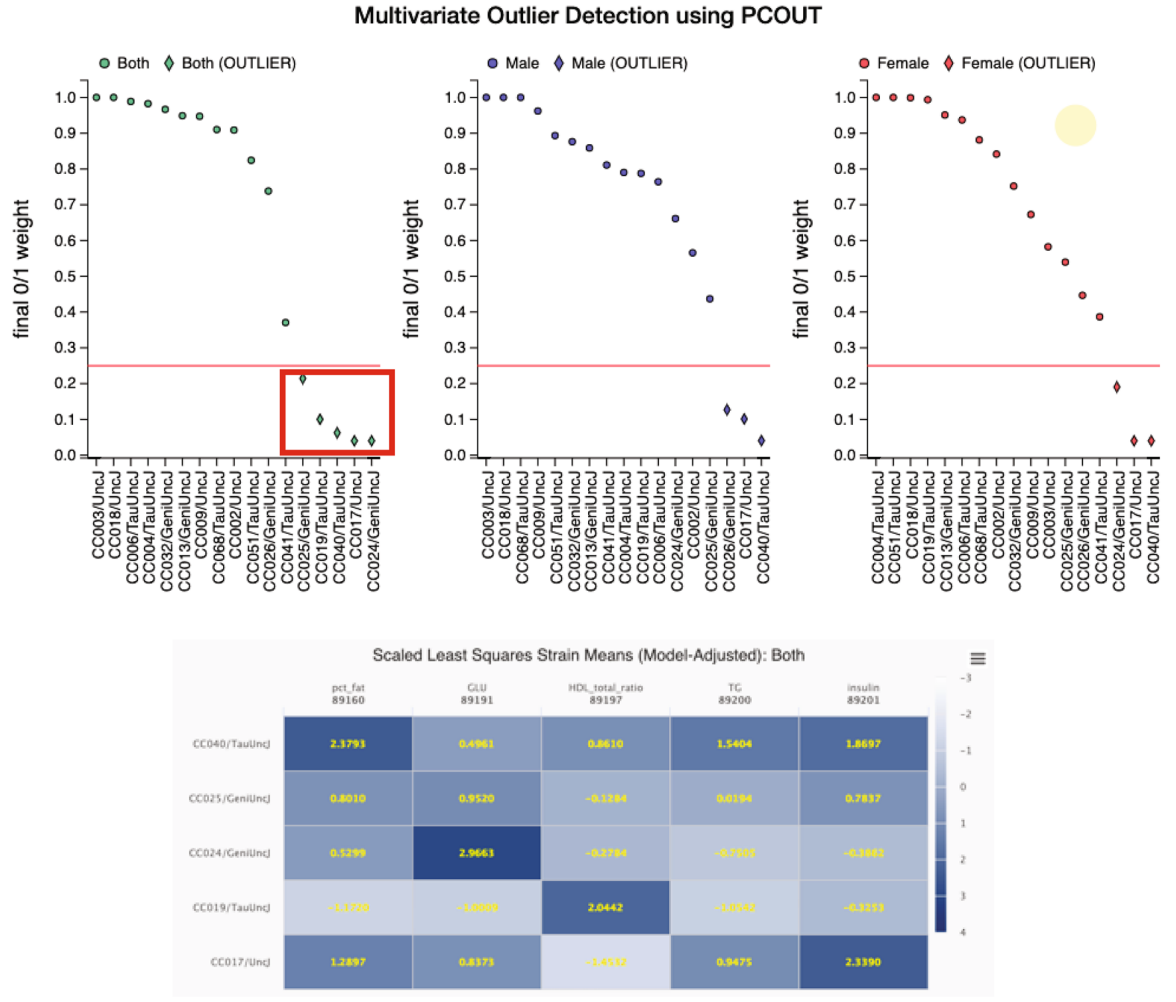
Multivariate outlier detection tool. Applied to a study of metabolic traits using the IMPC/KOMP phenotyping pipeline in Collaborative Cross strains. Strains below the red cut-off line are considered outliers. Selecting the outlier strains (click and drag resulting in the red box) in the first plot (both sexes) produces the heat map shown below of scaled least squares means to help users see at-a-glance phenotypic profiles for the selected strains. Data can be found at: https://phenome.jax.org/measures/89160, https://phenome.jax.org/measures/89191, https://phenome.jax.org/measures/89197, https://phenome.jax.org/measures/89200, https://phenome.jax.org/measures/89201

For syndromic conditions, or conditions in which multiple relevant measures may have been obtained to assess a latent dimension of phenotypic variation, one may wish to identify extreme strains based on multiple measures. For identifying multivariate outlier strains, MPD makes use of the R/PCOut procedure (Filzmoser et al. 2007). This tool is especially useful for identifying mouse models that are collectively extreme across a set of traits of interest. PCOut utilizes inherent properties of principal components decomposition and has been shown to be extremely efficient on higher dimension datasets, performing similarly on lower dimensional multivariate datasets as well. The method first computes semi-robust principal components, which are then used in determining distances for each observation, followed by calculations of weights for location and scatter outliers. Location and scatter weights are then combined to define a “Final 0/1 Weight” as plotted in the upper panel of Fig. 4. This plot is used for outlier identification, with strains approaching 0 signifying multivariate outliers (below the red line). Selecting strains (click and drag) will produce a table of color-coded scaled least squares strain means (the more extreme the color, the more extreme the outlier) as shown in Fig. 4 lower panel.

### Genotype effect sizes for a particular phenotype in an IMPC engineered mutant strain

**Fig. 5:**
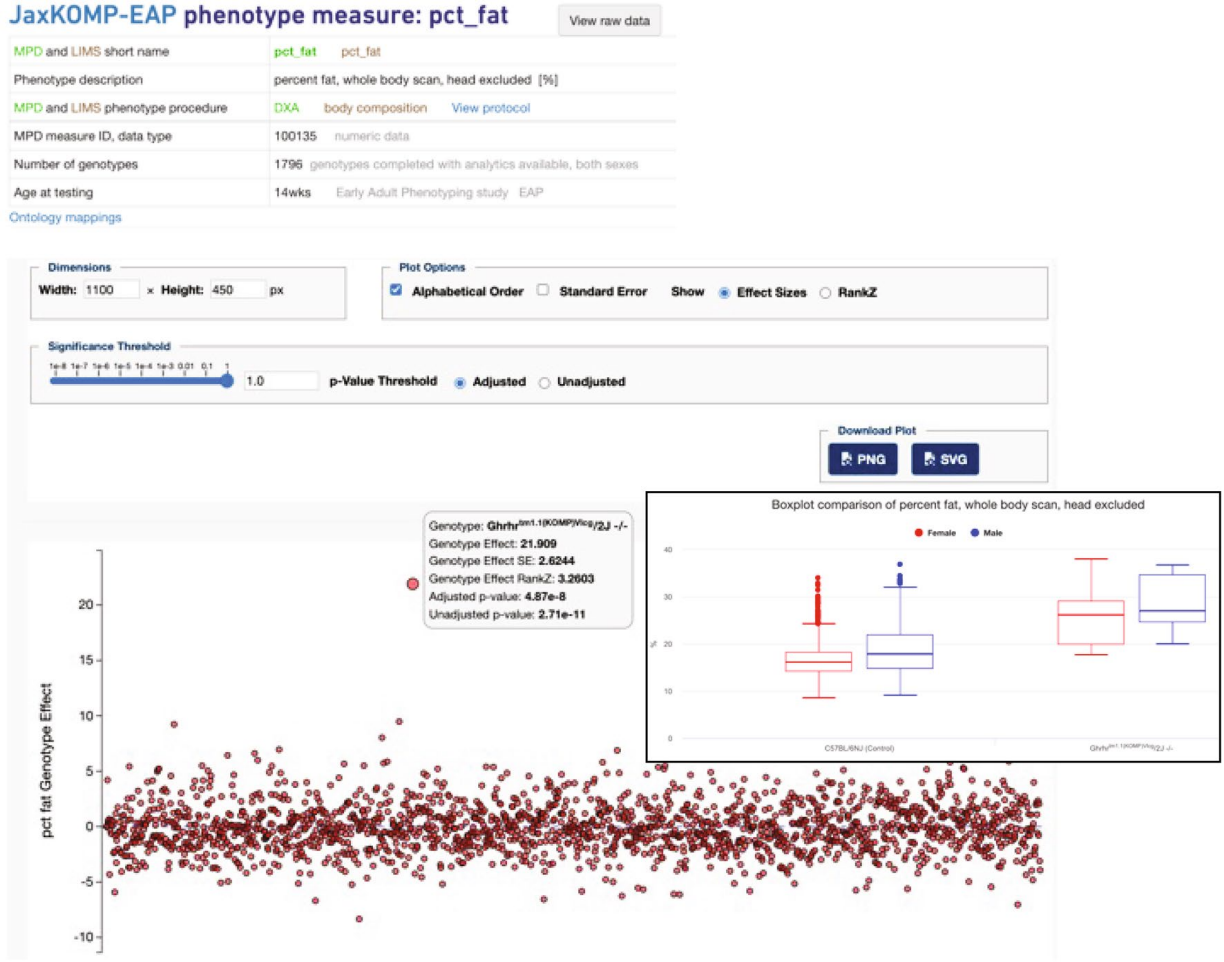
Genotype effect size plot for a particular phenotype in an IMPC engineered mutant strain. Each data point is a knockout strain. This is for the phenotype ‘percent fat, whole body scan’ for IMPC/JaxKOMP early adult pipeline data. Data were processed through the phenstat package with a soft window of temporally local controls and rank Z normalization to create a standard effect size for each strain and trait. P-value thresholds can be controlled through a slider as shown above the plot in the control panel. The default setting is p = 1 so that all data points are visible. Strain statistical values for the *Ghrhr* deletion mutant pop up by hovering over the data point. Clicking on that data point results in a box plot showing mutant versus control values (inset). Data can be found at: https://phenome.jax.org/komp/phenotypes/100135

Data from the IMPC/JaxKOMP center were processed through the phenstat package (Kurbatova et al. 2015) with a soft window of temporally local controls and rank Z normalization to create a standard effect size for each strain and trait. Control/mutant data can be viewed in several different ways. For example, in Fig. 5, the genotype effect sizes for all strains (genotypes) on any given measure can be viewed simultaneously (each data point represents a strain). Strains are in alphabetical order by default but can be ordered by magnitude as well (see option panel where deselecting ‘Alphabetical Order’ will result in ‘Magnitude Order’). A user can mouse-over data points to find out more information, including identifying the strain and viewing statistics as shown for the outlier strain representing the knockout for the gene *Ghrhr*. Clicking on the data point takes users to a box plot showing control/mutant data for quick comparison of the primary data (inset). Another important tool for viewing effect size correlations is the ‘Scatterplots and Correlations’ tool where users can choose multiple measures and run the analysis. Data points are genotype effect sizes so the user can easily see the degree to which pairwise measures are correlated (not shown).

### Phenotype profile for a particular genotype (control/mutant data)

**Fig. 6:**
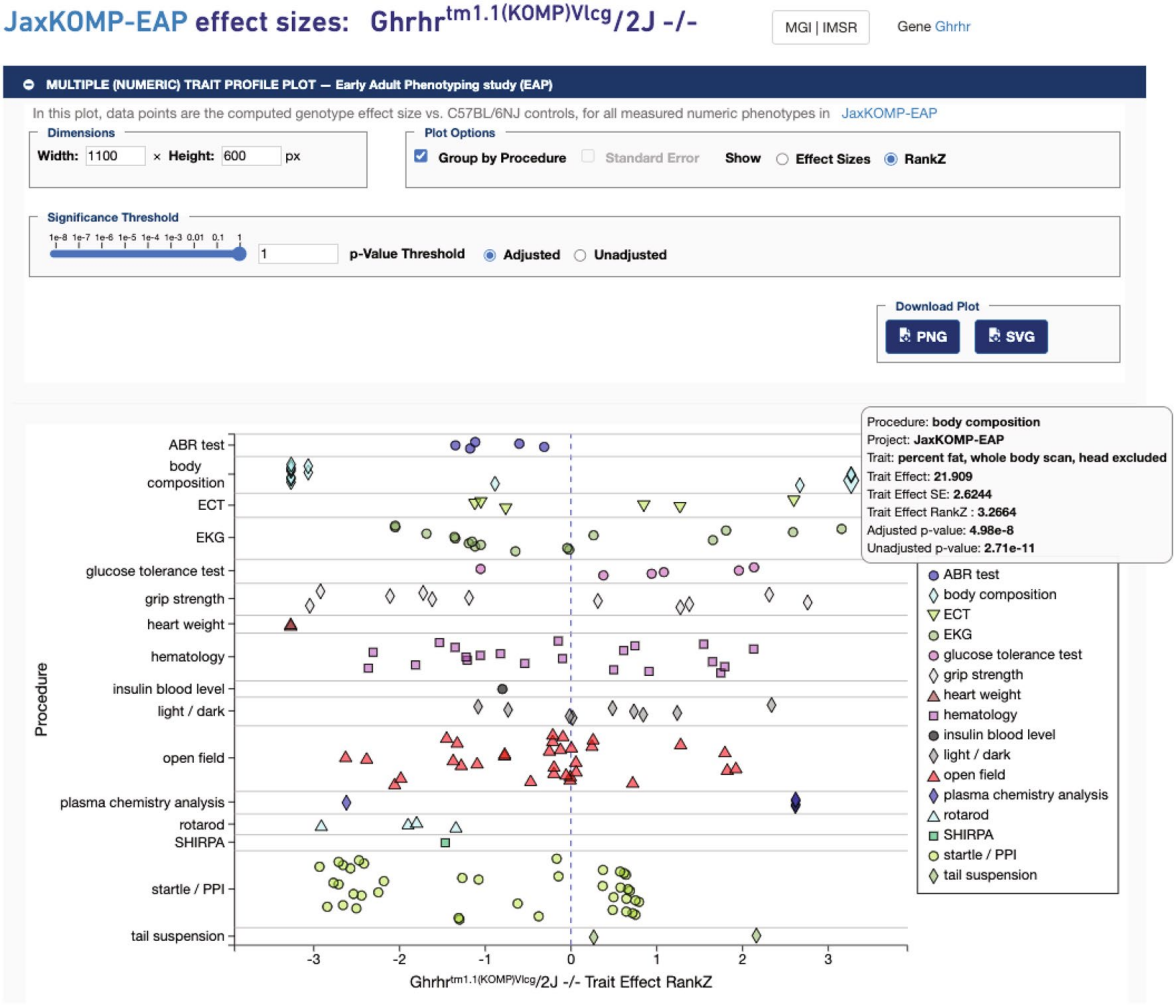
Phenotype profile plot for a particular genotype. Clicking on the gene name Ghrhr^tm1.1(KOMP)Vlcg^/2 J -/- on the box plot page (shown in inset of Fig. 5) or in the project’s Genotype table opens this view. All phenotypes measured for this knock-out strain are shown by default. Users can adjust the p-value significance threshold in the control panel above the plot. The plot will automatically update only with measures meeting the new criteria in p-value threshold (not shown). Hovering over the data point ‘percent fat, whole body scan’ reveals statistical values in a popup (shown). Data can be found at: https://phenome.jax.org/komp/genotypes/1945902?study=JaxKOMP-EAP

This tool plots trait effects (rank Z by default) for all phenotypic measures for a strain of interest. In this example, the phenotypic profile for the knockout of the gene *Ghrhr* is shown in Fig. 6. Mousing-over data points will provide more statistics (trait effect, trait effect p-value, trait effect rank Z, adjusted p-value, unadjusted p-value). In this case, ‘percent fat’ was moused-over which was the phenotype selected for the example in Fig. 5. Clicking on that data point will take the user to the same box plot as shown in the inset of Fig. 5.

### Pattern matching to find strains with multi-trait profiles

**Fig. 7:**
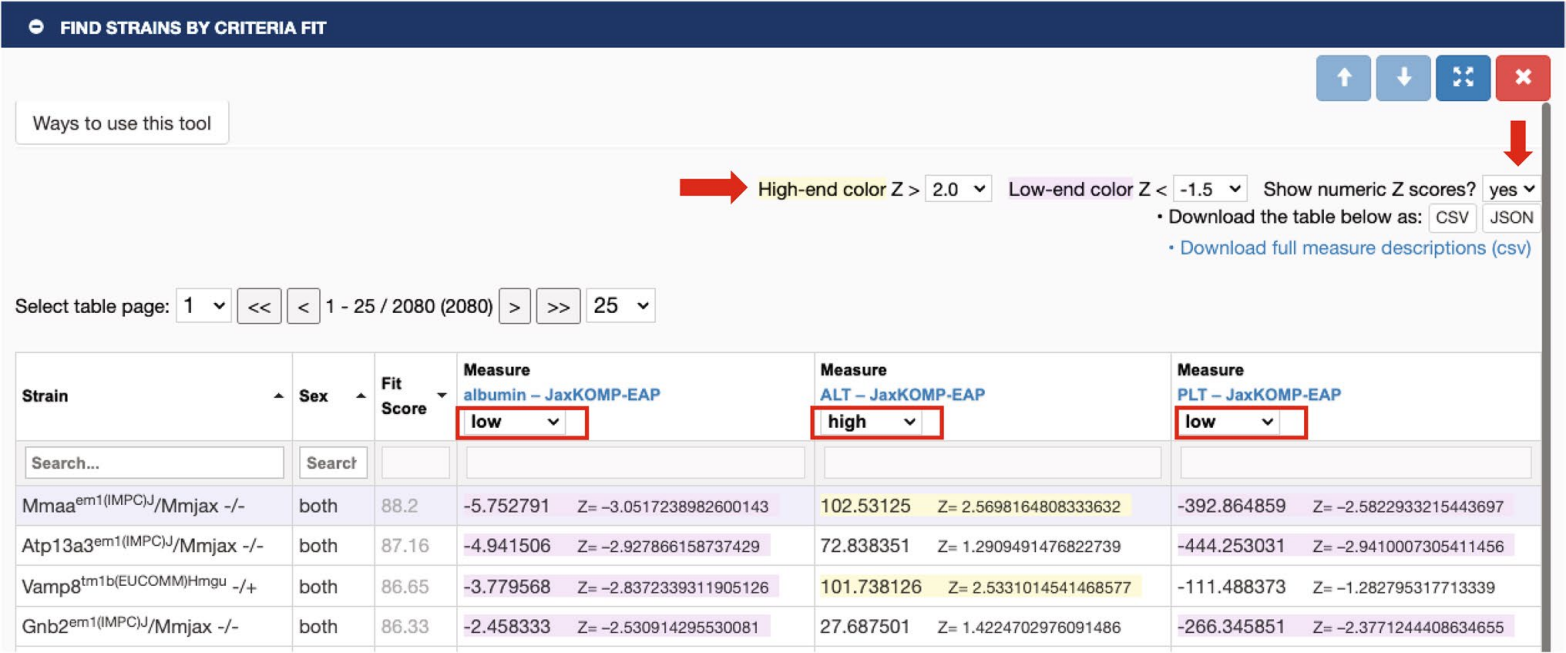
Find Strains by Criteria Fit Tool. Users select their measures of interest and then select this tool. In this example, we have chosen albumin, alanine transaminase (ALT), and platelet count (PLT). Users then select the Z-score threshold (right red arrow) and their criteria for each measure. Here we have chosen low, high, low, respectively (boxed in red). The results table is shown. Users may then opt to see Z-scores displayed (down red arrow) which give an indication of how extreme the measures are. In this example only one strain out of over 2000 meets our criteria, the knockout for gene *Mmaa*. Data can be found at: https://phenome.jax.org/komp/phenotypes/100151, https://phenome.jax.org/komp/phenotypes/100169, https://phenome.jax.org/komp/phenotypes/100291

Another important tool for strain panels and IMPC data are ‘Find Strains by Criteria Fit’ where a user can select multiple phenotypes of interest and ask to see outlier strains based on user-selected Z-score thresholds as shown in Fig. 7. Each result comes with a best-fit score and results are sorted by default on this score. This tool provides a means to choose mouse mutant models that manifest a user’s particular combination of phenotypes of interest, for example a set of traits that appear in syndromic disease.

### GenomeMUSter

The integration of variant data across strains provides a comprehensive resource with which to interpret and utilize the effects of variation observed across the large numbers of extant inbred mouse strains. Numerous genotype datasets have been merged so far, including MPD legacy datasets (Bogue et al. 2023), B6Eve (Sarsani et al. 2019), Collaborative Cross strains (Srivastava et al. 2017), recent BXD data (Ashbrook et al. 2022 and Sasani et al. 2022), recent SNP data on 42 inbred strains (Arslan et al. 2023), and Sanger data (Keane et al. 2011). GenomeMUSter currently includes typed, sequenced, and imputed allelic states for 657strains at 108.6 + million locations, with more expected to be included as datasets become available from members of the research community. This analytical resource and the accompanying user interface (UI) and API allow browsing, visualizing, filtering on genotype confidence level, and downloading SNP data. Additional functionality will be added in the near future that will allow a user to compare variation across user-defined strain groups and will enable filtering based on functional annotations. The GenomeMUSter resource will be used for GWAS Meta-analysis (next section). A quick link for GenomeMUSter is https://muster.jax.org.

### GWAS meta-analysis

To examine the shared and distinct genetic regulation of multiple user-selected measures and to improve power for mapping, one can run a GWAS meta-analysis on that data (not shown) using METASOFT, developed in the Eskin Lab at UCLA (Han and Eskin 2011, 2012). Users can select individual measures which are collected and saved in a measure set, or they can aggregate data by ontology term or other metadata to define their measure set. Results for the meta-analysis are presented as a single Manhattan plot, and SNPs can be selected for subsequent P-M Plots to evaluate the individual contribution of the study to the variant association score (Kang et al. 2016). Meta-analysis results are also visualized using Forest Plots, which provide an observed effect size, confidence interval and weight for each measure along with an overall pooled effect size across all measures (Kang et al. 2016). Fuji plots are genome-wide Circos plots which provide a top-down whole-genome view of variant effects annotated with traits where the effect exists (m-value > 0.9). Mouse genes and their orthologous human genes associated with these variants through the VariantGraph (Reynolds et al. 2021) are annotated to each variant. Orthologous human genes are presented in tabular format, providing powerful cross-species comparisons.

## Implementation

The MPD ecosystem has been migrated to Google Cloud Platform (GCP) where the following applications are deployed using a Kubernetes cluster: the MPD web application, the Study Intake Platform (SIP), the MPD analysis service, and the METASOFT service. The MPD web application is a legacy Python Flask application, rendering pages using Jinja2 templates in HTML and JavaScript. SIP, a Python Flask RESTplus application, provides users access through an Angular web application and program access via REST endpoints. Both of these applications use a PostgreSQL database which is hosted in the cloud using CloudSQL. The MPD Analysis Server is also a Python RESTplus web service application. It has analytics implemented in Python and leveraging Pandas and via rpy2 to call an R-based analysis package which is implemented by data analysts and statisticians on the MPD team. The METASOFT service is a Java Spring Boot web service which submits long running jobs to a workflow managed by Temporal.io. We have been re-architecting all legacy aspects of the application and implement it using an Angular client with PrimeNG components and styling and which uses REST API services.

GCP Kubernetes applications are deployed in two clusters: 1) development and testing cluster and 2) production cluster (has a staging and production instance). MPD developers can deploy updates to the development and testing environments. All code goes through a code review process prior to being deployed to the testing cluster where our Software Quality Assurance team tests all software prior to deployment to the staging environment for final review and testing before release to our production environment.

## Submitting data

We accept strain means data but prefer per-animal data as it is amenable to more rigorous statistical analyses and so that researchers can drill down to individual data quickly. Our recently released Study Intake Platform (SIP) is ready for data contributors to upload and annotate their own data. As domain experts, they are often the most capable of appropriately annotating with ontology terms. Data will be subject to MPD curatorial review. If interested in submitting data to MPD, simply go to the SIP homepage at https://studyintake.jax.org. Registration is required so that viewing/editing privileges can be granted while keeping the study private until the contributor is ready to go public. On the SIP homepage, click on the ‘?’ icon in the left menu to access detailed guidelines on how to format datasets and load data and metadata. There is also a FAQ to help answer your questions. Data contributors can contact us at phenome@jax.org for support in curation or in the use of the application. Data contributors can also submit data and supporting documentation via email attachment, in which case, a professional curator will process the study. Contact us at phenome@jax.org for this option. Historically, most MPD studies have been submitted via email, but we expect more studies to be submitted through SIP in the future.

## Conclusions

Through an expanded set of analytic tools and data resources, we provide users new avenues for data integration and interpretation across multiple genes, traits, and populations. Data from the MPD repository are available for use by other tool sets and will be provided through an increasingly dynamic and FAIR-compliant API, and the flexible user interface enables rapid access to exploratory analyses backed by rigorous analytic tools. We continue to move toward fully traceable and repeatable analyses, and interoperation with other data services to expand the backdrop of information used to interpret and contextualize mouse phenotypic diversity. Users of the MPD resource now have access to a wealth of new functions to provide insights into phenotypic diversity and its relation to human health and disease.

## Data Availability

API endpoints are available for programmatic access to phenotype data, metadata, and analytics results (JSON or csv). MPD endpoints are available through an API Gateway with endpoint and parameter documentation. For more information see https://phenome.jax.org/about/api. Bulk data downloads are available at https://phenome.jax.org/downloads in csv format. See figure legends for specific data accessed for these examples.

## References

[CR1] Arslan A, Fang Z, Wang M (2023). Analysis of structural variation among inbred mouse strains. BMC Genom.

[CR2] Ashbrook DG, Sasani T, Maksimov M et al (2022) Private and sub-family specific mutations of founder haplotypes in the BXD family reveal phenotypic consequences relevant to health and disease. 2022.04.21.489063

[CR3] Bandrowski AE, Martone ME (2016). RRIDs: a simple step toward improving reproducibility through rigor and transparency of experimental methods. Neuron.

[CR4] Basilico B, Ferrucci L, Ratano P (2022). Microglia control glutamatergic synapses in the adult mouse hippocampus. Glia.

[CR5] Bogue MA, Ball RL, Philip VM (2023). Mouse Phenome Database: towards a more FAIR-compliant and TRUST-worthy data repository and tool suite for phenotypes and genotypes. Nucleic Acids Res.

[CR6] Brommage R, Ohlsson C (2019). High fidelity of mouse models mimicking human genetic skeletal disorders. Front Endocrinol (Lausanne).

[CR7] Cacheiro P, Haendel MA, Smedley D, International Mouse Phenotyping Consortium and the Monarch Initiative (2019). New models for human disease from the International Mouse Phenotyping Consortium. Mamm Genome.

[CR8] Churchill GA, Gatti DM, Munger SC, Svenson KL (2012). The Diversity Outbred mouse population. Mamm Genome.

[CR9] da Silva-Buttkus P, Spielmann N, Klein-Rodewald T (2023). Knockout mouse models as a resource for the study of rare diseases. Mamm Genome.

[CR10] Filzmoser P, Maronna R, Werner M (2007). Outlier identification in high dimensions.

[CR11] Groza T, Gomez FL, Mashhadi HH (2023). The International Mouse Phenotyping Consortium: comprehensive knockout phenotyping underpinning the study of human disease. Nucleic Acids Res.

[CR12] Han B, Eskin E (2011). Random-effects model aimed at discovering associations in meta-analysis of genome-wide association studies. Am J Hum Genet.

[CR13] Han B, Eskin E (2012). Interpreting meta-analyses of genome-wide association studies. PLoS Genet.

[CR14] Hayamizu TF, Mangan M, Corradi JP (2005). The Adult Mouse Anatomical Dictionary: a tool for annotating and integrating data. Genome Biol.

[CR15] Higgins K, Moore BA, Berberovic Z (2022). Analysis of genome-wide knockout mouse database identifies candidate ciliopathy genes. Sci Rep.

[CR16] Jaljuli I, Kafkafi N, Giladi E (2023). A multi-lab experimental assessment reveals that replicability can be improved by using empirical estimates of genotype-by-lab interaction. PLoS Biol.

[CR17] Kafkafi N, Benjamini Y, Sakov A (2005). Genotype-environment interactions in mouse behavior: a way out of the problem. Proc Natl Acad Sci USA.

[CR18] Kafkafi N, Golani I, Jaljuli I (2017). Addressing reproducibility in single-laboratory phenotyping experiments. Nat Methods.

[CR19] Kang EY, Park Y, Li X (2016). ForestPMPlot: a flexible tool for visualizing heterogeneity between studies in meta-analysis. G3 (Bethesda).

[CR20] Keane TM, Goodstadt L, Danecek P (2011). Mouse genomic variation and its effect on phenotypes and gene regulation. Nature.

[CR21] Kurbatova N, Mason JC, Morgan H (2015). PhenStat: a tool kit for standardized analysis of high throughput phenotypic data. PLoS ONE.

[CR22] Lin D, Crabtree J, Dillo I (2020). The TRUST Principles for digital repositories. Sci Data.

[CR23] Nadon NL, Strong R, Miller RA, Harrison DE (2017). NIA interventions testing program: investigating putative aging intervention agents in a genetically heterogeneous mouse model. EBioMedicine.

[CR24] Park CA, Bello SM, Smith CL (2013). The Vertebrate Trait Ontology: a controlled vocabulary for the annotation of trait data across species. J Biomed Semant.

[CR25] Percie du Sert N, Hurst V, Ahluwalia A (2020). The ARRIVE guidelines 2.0: updated guidelines for reporting animal research. PLoS Biol.

[CR26] Peterson KA, Murray SA (2022). Progress towards completing the mutant mouse null resource. Mamm Genome.

[CR27] Reynolds T, Johnson EC, Huggett SB (2021). Interpretation of psychiatric genome-wide association studies with multispecies heterogeneous functional genomic data integration. Neuropsychopharmacology.

[CR28] Sarsani VK, Raghupathy N, Fiddes IT (2019). The genome of C57BL/6J “Eve”, the Mother of the Laboratory Mouse Genome Reference Strain. 3G (Bethesda).

[CR29] Sasani TA, Ashbrook DG, Beichman AC (2022). A natural mutator allele shapes mutation spectrum variation in mice. Nature.

[CR30] Smith CL, Eppig JT (2012). The Mammalian Phenotype Ontology as a unifying standard for experimental and high-throughput phenotyping data. Mamm Genome.

[CR31] Srivastava A, Morgan AP, Najarian ML (2017). Genomes of the mouse collaborative cross. Genetics.

[CR32] Stefancsik R, Balhoff JP, Balk MA (2023). The Ontology of Biological Attributes (OBA)—computational traits for the life sciences. Mamm Genome.

[CR33] Svenson KL, Gatti DM, Valdar W (2012). High-resolution genetic mapping using the Mouse Diversity outbred population. Genetics.

[CR34] Viterbi A (1967). Error bounds for convolutional codes and an asymptotically optimum decoding algorithm. IEEE Trans Inf Theory.

[CR35] Wilkinson MD, Dumontier M, Aalbersberg IJJ (2016). The FAIR Guiding Principles for scientific data management and stewardship. Sci Data.

